# Photoacoustic Permeability Detection to Both Water Vapor and Artificial Tears of PVDF Transfer Membranes as a Piezoelectric and Ferroelectric Polymer

**DOI:** 10.3390/polym18081009

**Published:** 2026-04-21

**Authors:** Oscar E. Aguilar-Mejía, Lilia I. Olvera-Cano, Jose J. A. Flores-Cuautle, Alfredo Cruz-Orea, Ernesto Suaste-Gómez

**Affiliations:** 1 Cinvestav, Department of Electrical Engineering, Section Bioelectronics, Av. IPN #2508 Col. San Pedro Zacatenco, Mexico City 07360, Mexico; oscar.aguilarm@cinvestav.mx; 2 Cinvestav, Department of Physics, Av. IPN #2508 Col. San Pedro Zacatenco, Mexico City 07360, Mexico; lilia.olvera@cinvestav.mx (L.I.O.-C.); alfredo.cruzorea@cinvestav.mx (A.C.-O.); 3 Postgraduate Studies and Research Division, Technological Institute of Orizaba, Orizaba 94320, Mexico; jflores_cuautle@hotmail.com; 4 Ministry of Science, Humanities, Technology, and Innovation-SECIHTI, Mexico City 03940, Mexico

**Keywords:** photoacoustic detection, permeability measurement, water vapor, artificial tears, PVDF

## Abstract

Photoacoustic configuration studies were performed to measure the water vapor permeability of the polymeric PVDF transfer membranes with a pore size of 0.45 µm and a thickness of 117 and 119 µm, taking advantage of the characteristic that the polyvinylidene difluoride (PVDF), once polarized by the corona poling, creates a piezoelectric polymer. Polymeric membrane measured polarized as a piezoelectric polymer and an unpolarized ferroelectric polymer. Polarized and non-polarized PVDF membranes were developed for the two experimental photoacoustic detection of permeability tests; the first one was used to measure the humidity of bi-distilled water, and the second one was characterized with artificial tears. The obtained results show that PVDF membrane has different permeability coefficient for water and artificial tears, and at the same time, pores in the tested membranes change sizes depending on the liquid used. The results of the permeability and pore size of the PVDF membranes provide insight into vapor transport mechanisms that may inform the future development of humidity sensors.

## 1. Introduction

Photothermal (PT) techniques are based on the optical absorption of a sample due to a modulated light beam impinging on that sample, followed by a non-radiative de-excitation process, which generates periodic heating and, subsequently temperature fluctuation in the sample; from these phenomena, different PT techniques have been developed for sensing these fluctuations [1]. Among PT techniques, Photoacoustic Spectroscopy (PAS) is highlighted by its diverse applications in the optical characterization of materials. In a conventional PAS experimental setup, the sample is enclosed in an air-tight cell and exposed to a modulated light beam [1]. As a result of sample periodic heating, due to light absorption, the air pressure inside the cell oscillates at the light modulation frequency and can be detected by a microphone or a piezoelectric sensor coupled to the cell [1,2]. PAS has been used in the thermal characterization of a great variety of materials, ranging from polymers [3,4], semiconductors [5,6], foodstuffs [7,8], and biological specimens [9,10]. Several approaches have been developed to study vapor transport and humidity sensing in porous materials, including electrical (capacitive and resistive) sensors, gravimetric methods, and optical techniques such as fluorescence-based sensing. For in-stance, fluorescence-based methods have demonstrated high sensitivity in monitoring transport processes in porous systems [11]. However, these techniques often require transparent samples, labeling, or complex optical configurations. In contrast, the photoacoustic approach offers several advantages, including applicability to opaque and scattering materials, non-contact detection, sensitivity to sub-surface transport processes, and robustness under varying environmental conditions. On the other hand, Polyvinylidene fluoride (PVDF) stands out not only because of its piezoelectric and pyroelectric properties, but also because PVDF transfer membrane has been researched due to its hydrophobic properties. It is essential in the development of a humidity sensor because it prevents the storage of water molecules in the sensing material, and at the same time, it allows water vapor to flow fluently.

PVDF has been used as a capacitive sensor. Capacitive humidity sensors exhibit several advantages compared with resistive humidity sensors, such as linear responses, fast response time, and low hysteresis [12,13]. Different techniques have been used for the thermal and optical characterization of biopolymers and polymers as PVDF membranes [14,15,16,17,18,19,20,21]. Another interesting property of PVDF is the biocompatibility of the material; thus, PVDF has been used as a sensor in the biomedical field and in biological imaging applications [13,15,16]. Accordingly, Sigma Aldrich develops a PVDF syringe (Sigma Aldrich WHA67771302) and channel (Sigma Aldrich WHA77073000) filters and PVDF Western blotting membranes (Sigma Aldrich GE10600023) to identify proteins.

Hernández et al. [12] have reported the PVDF relative humidity response, showing that the PVDF membrane responds faster than a DTH11, which is a well-known humidity electronic sensor. Nevertheless, the PVDF transfer membrane also responds to direct moisture changes. Martínez et al. [22] described the simulated development of a system based on PVDF ferroelectric polymeric transfer membrane as an ocular moisture sensor to support the diagnosis of dry eye. Despite these advances, the use of photoacoustic techniques to investigate vapor transport in PVDF membranes—particularly under conditions relevant to biomedical applications such as artificial tear exposure—remains relatively unexplored. In this paper, a photoacoustic (PA) configuration is employed to detect the water vapor permeability of polymer films. In the employed experimental setup, the PA heat source is located at the cell’s optical entrance window rather than at the sample–transducer gas interface. In this way, it is claimed that only the sample permeability property is experimentally accessed. This experimental arrangement is applied to measure the water vapor permeability of the PVDF polymeric transfer membrane. Taking advantage of the characteristic that PVDF, once polarized by corona poling, creates a piezoelectric polymer [23]. Photoacoustic configuration studies were performed to detect the water vapor permeability of PVDF polarized as a piezoelectric polymer and an unpolarized ferroelectric polymer [23].

## 2. Materials and Methods

### 2.1. PVDF Membranes and Artificial Tears

Two porous PVDF polymeric transfer membranes with 0.45 µm pore size and a thickness of 117 and 119 µm (P2563-10 EA, Sigma Aldrich, St. Louis, MI, USA) manufactured by Millipore (Cork, Irland) with catalog number IPVH15150 were considered for the experimental test; the first one was used to measure the moisture of bi-distilled water and the second one was characterized with artificial tears (Moisture Eyes, Bausch and Lomb, Pelham Road, Greenville, SC, USA). Both samples were obtained from the same original membrane sheet, which was cut into two pieces to minimize variability in material properties. Bausch and Lomb Moisture eyes are lubricant eye drops compound by two lubricants, glycerin (0.3%) and propylene glycol (1%), that moisturize dry eyes and help prevent further irritation. It is important to note that this photoacoustic configuration was carried out to detect the water vapor permeability of polarized (piezoelectric polymer) and unpolarized (ferroelectric polymer) PVDF. To obtain the PVDF piezoelectric polymer, the most highly polar phase is on the β-phase, whose unit cell consists of two all-trans chains packed with their dipoles pointing in the same direction [23].

### 2.2. Corona Poling

As reported in the literature, corona poling favors transition to β-phase in PVDF; thus, enhancing piezoelectric properties [24]. The polarizing process was performed by corona poling, consisting in placing the membrane sample in a grounded-copper plate under an electric field of 15 kV through a needle. The needle was placed 15 cm from the sample and the electrical field was maintained for 2 min per sample. Because the literature reports that this poling procedure results in PVDF polarization and β-phase development, no further measurements were performed for PVDF poling [25,26].

### 2.3. Photoacoustic Experimental Setup

**Figure 1:**
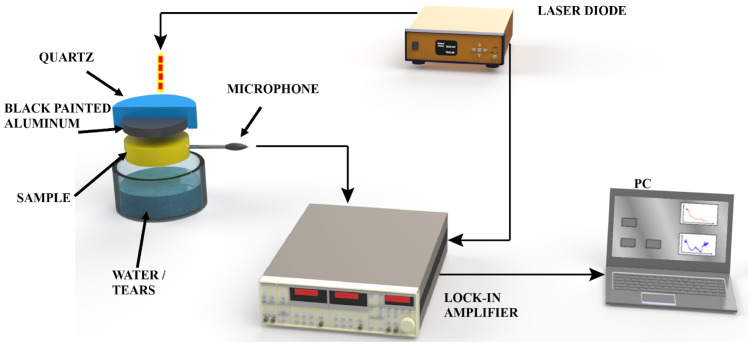
Diagram of the experimental setup for photoacoustic detection of water vapor permeability in solid samples.

Figure 1 shows the experimental arrangement for photoacoustic detection of water vapor permeability in solid samples. It consists of a cylindrical PA cell machined on a 10 mm thick PVC plate. The cell is closed at the outer front side by an optical window through which the modulated light beam from a semiconductor laser is directed [27,28]. The inner side of this optical window, adjacent to the transducer gas of the cell, has a thin (17 µm thick) aluminum (Al) foil stuck to it with the help of a thin layer of silicone vacuum grease. This Al foil, whose side in contact with the optical window is painted black, works essentially as a light absorber. The back end of the PA cell is closed with the sample. The air pressure fluctuations within the PA cell, following the periodic heating caused by the absorption of the incoming light beam, are sensed by an electret microphone connected to the cell air of the chamber through a 1 mm wide duct. The outer sample surface is exposed to a relative humidity-controlled environment. This consists of a PVC cup filled with distilled water to ensure a 100% relative humidity atmosphere and silica gel to create a near 0% relative humidity atmosphere. These conditions provide reproducible humidity gradients suitable for comparative analysis, although they do not represent absolute humidity calibration. The cup is filled up to the point of leaving around a 10 mm air gap between the top level of the water or silica gel, and the sample [27,28].

The output voltage from the microphone was connected to a lock-in amplifier in which the PA signal amplitude and phase were recorded. The samples used in our measurements were porous PVDF polymeric transfer membranes. These samples were placed between two centered sheets of virgin polystyrene, with a 1 mm hole diameter in their centers, to allow the water vapor to pass through the sample only in this fixed area. The experimental procedure for the PA measurements consisted of exposing the back sample surface to a given relative humidity environment and recording the corresponding PA signal as a function of time. All measurements were carried out at a fixed modulation frequency of 17 Hz, which was selected to ensure stable signal detection and an adequate signal-to-noise ratio [27,28].

### 2.4. SEM Imaging and Image Processing

Scanning electron microscopy (SEM) images of the PVDF membranes were obtained using a JEOL JSM-7401f (Tokyo, Japan) microscope with the following parameters: $V_{acc}=1.0\;\mathrm{kV}$, WD=15mm, 1000× (scale bar: 10μm) and 20,000× (scale bar: 1μm). Samples were analyzed immediately after exposed to water and tear vapor. No sample preparation was performed before SEM analysis.

SEM micrographs were analyzed using DiameterJ (ImageJ/FIJI), 1.51w version [29,30]. DiameterJ allows for the extraction of structural parameters, including pore size distribution, fiber orientation, and intersection density, providing a comprehensive characterization of fibrous networks. The membrane image analysis started with image pretreatment that comprised noise removal, and image smoothing; those steps were performed using the predefined software levels. DiameterJ allows the selection of a Region of Interest (ROI). The entire image was selected as ROI, only excluding the microscopy labels. Because the pore analysis requires determining fiber boundaries, the Auto Threshold software feature was applied for segmenting images. Once images are binary, clusters of black pixels (holes) are identified; clusters were labeled as pores based on circularity which was set between 0.5 to 1. Finally, the pore area was determined using the number of pixels comprising each cluster and physical image dimensions. The pore size reflects the microstructural features of the membrane and plays a key role in determining its permeability and overall transport behavior.

## 3. Results and Discussion

**Figure 2:**
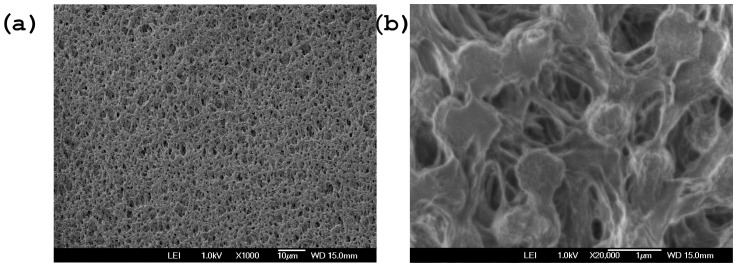
SEM Micrographs of PVDF polymeric transfer membrane with a pore size of 0.45 µm and a thickness of 117 µm at magnification (**a**) 1000×, (**b**) 20,000×.

Figure 2 shows SEM images of PVDF polymeric transfer membrane with a pore size of 0.45 µm and a thickness of 117 µm with magnifications 1000× and 20,000×. It should be noted that SEM analysis was used as qualitative support for the observed transport behavior. Because the piezoelectric and pyroelectric nature of the PVDF samples the quality of SEM images is affected, the electron beam induces pyroelectric charges due to the inelastic scattering. On the other hand, the localized electric field induced by the electron beam can provokes mechanical deformations due to converse piezoelectric effect, which in turns deforms the sample during the scan.

**Figure 3:**
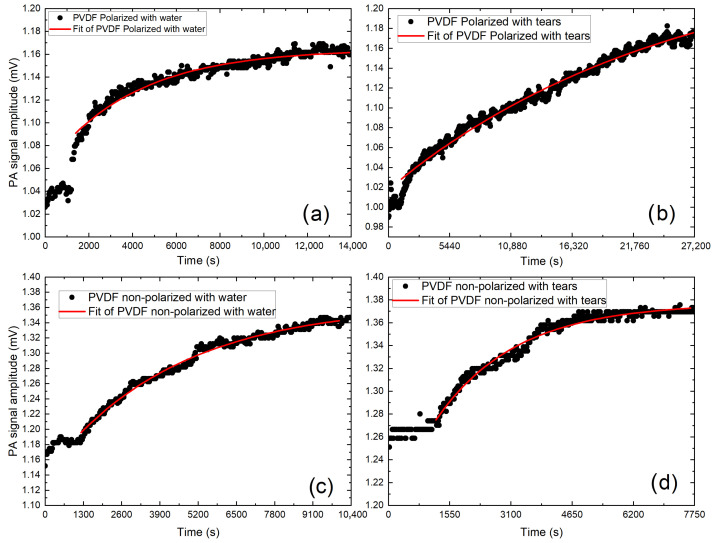
The black dots represent the amplitude of the photoacoustic signal over time for the water vapor diffusion experiments (**a**,**c**) and tear vapor diffusion experiments (**b**,**d**) with polarized PVDF (**a**,**b**) and non-polarized PVDF (**c**,**d**). The red solid line shows the best fit of Equation (1) to the experimental data in all cases.

Figure 3 shows typical PA signals as a time function during a dry–wet transition. The sample is initially exposed to silica gel in the PVC cup for 20 min for a drier environment. Subsequently, it is replaced with bi-distilled water in the PVC cup to have a 100% relative humidity environment, and the PA signal is monitored until it achieves stability. The time for stabilization depends on the sample permeability to the water vapor diffusion. The time evolution of the PA signal amplitude shown in Figure 3 results from the diffusion of water vapor through the sample and is described by first-order kinetics. That is, we can describe the time evolution of the PA signal by

(1) $$S=S_{0}+S_{1}\left(1-e^{-\frac{t-t_{0}}{t_{d}}}\right)$$

where $S_{0}$ represents the initial amplitude of the PA signal when the sample is exposed to the dry atmosphere, $t_{0}$ is the time of change of the relative humidity of the atmosphere, $S_{1}$ is the excursion of the PA signal until it reaches the saturation value at the new ambient temperature and $t_{d}$ is the sample’s water vapor diffusion time, given by $t_{d}=\frac{l_{s}^{2}}{2D}$. Here, $l_{s}$ is the sample’s thickness and *D* is the water vapor’s diffusion coefficient [20,21].

Fitting procedure was employed to determine the time delay and diffusion coefficients for the analyzed samples. The experimental data were fitted to Equation (1) and diffusion coefficient values are presented in Table 1. At the beginning, the measurements showed that the signal was noisy, and a signal-stabilization time was considered. Therefore, all fittings started at 1240±110 s, and reached a $R^{2}$ over 0.86.

After photoacoustic measurements, SEM images were acquired and pore area analysis was performed on SEM micrographs using DiameterJ (ImageJ/FIJI) [29].

**Figure 4:**
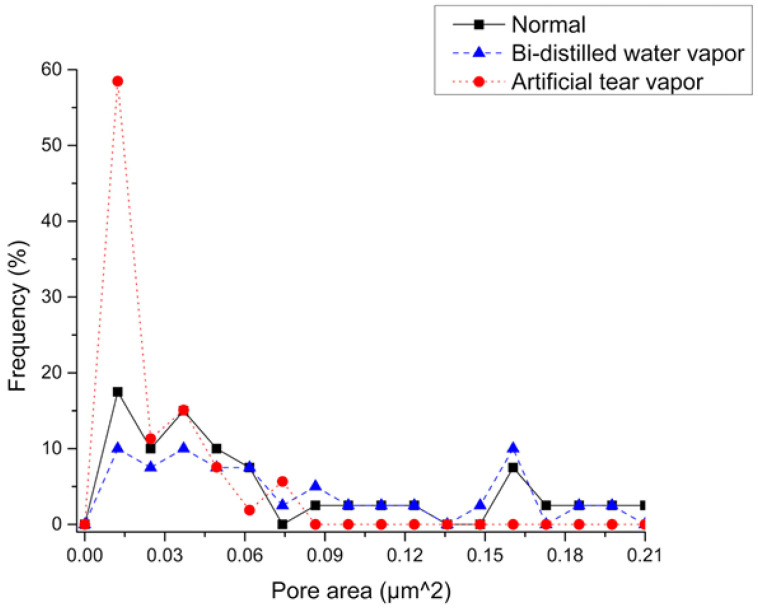
Pore size frequency of normal, polarized PVDF transfer membrane after bi-distilled water vapor exposure and polarized after artificial tear vapor exposure.

The vapor diffusion coefficient (*D*) is lowest in polarized PVDF that is subject to tears, as shown in Table 1. The decrement in *D* is a direct result of the pore size reduction due to the poling process. Pores in the membrane are subject to electrostatic force during poling process [31]; thus, Maxwell stress leads to pore size reduction as confirmed by Figure 4. PVDF membranes exhibit high water absorption capabilities [32], which, combined with the pore size reduction, led to the largest water vapor diffusion time ($t_{d}$).

**Figure 5:**
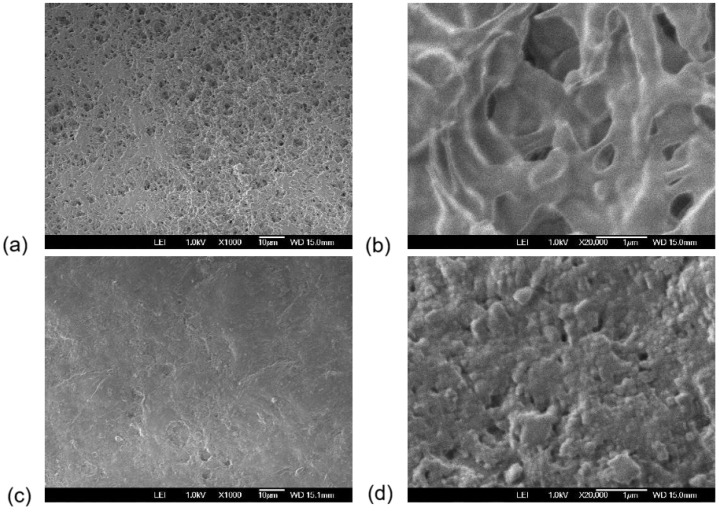
SEM micrographs of a polarized PVDF transfer membrane at magnifications of (**a**) 1000× and (**b**) 20,000× after exposure to water vapor and (**c**) 1000× and (**d**) 20,000× after exposure to tear vapor, illustrating permeability effects.

Figure 5a,b show visible pores in the structure of polarized PVDF exposed to bi-distilled water vapor, which can be associated with the hydrophobic properties of the PVDF transfer membrane, as previously reported [22]. Those images were obtained at magnifications of 1000× and 20,000×, respectively. In contrast, Figure 5c,d show the polarized PVDF exposed to tear vapor at the same magnifications.

**Figure 6:**
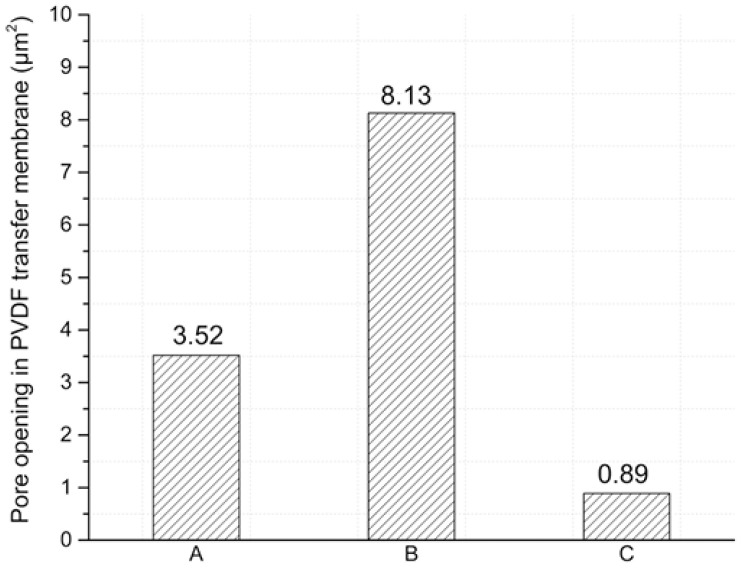
The total area of the opening in the PVDF transfer membrane under normal state conditions (A), after exposure to bi-distilled water vapor (B), and after exposure to vapor from artificial tears (C).

Figure 6 presents the total aperture area of the transfer membranes in their normal state and after exposure to the vapors. A quantitative reduction in pore size after exposure to artificial tear vapor and an increase in pore size after exposure to bidistilled water vapor can be observed. This is further illustrated in Figure 6, where the largest number of small pore areas appear in the PVDF transfer membrane exposed to artificial tear vapor. These micrographs are consistent with the results obtained in Table 1, indicating that PVDF film exposed to artificial tear vapor exhibits lower permeability compared to PVDF exposed to bi-distilled water vapor. The obtained water vapor diffusion coefficients are similar to those reported in the literature in several polymeric membranes [7,27,28,33].

The change in membranes pore size change can be related to the hydrophobic behavior of the membranes. The water vapor provokes a repulsive force over the pore boundaries which, in combination with the elastic properties of the membrane, increases the pore size. The force acting on the membrane pores is such that it increases only the pores with reduced areas, while pores larger than 0.03 $\mu m^{2}$ maintain their initial size. SEM images show differences in pore sizes after membranes have been exposed to different liquids resulting in differing vapor permeability. The change in vapor permeability opens up research areas because of the selective permeability membrane properties.

## 4. Conclusions

Studies on water and artificial tear vapor permeability in PVDF membranes using the photoacoustic technique have yielded significant findings. When exposed to different vapors, PVDF membranes polarized as piezoelectric polymers exhibited different behaviors. For water vapor permeability, polarized membranes showed a higher diffusion coefficient than artificial tear vapor permeability, indicating a more remarkable ability to allow the passage of water vapor. This is corroborated by the pore structure visible in polarized membranes after exposure to water vapor. In contrast, polarized membranes exhibited a much lower diffusion coefficient when exposed to artificial tear vapor. Scanning electron micrographs revealed a reduction in pore size in the membranes after exposure to tear vapor, explaining the significantly lower permeability observed. These results highlight that the polarization of PVDF membranes notably influences their permeability characteristics depending on the type of vapor. Polarized membranes’ greater water vapor permeability can be utilized to design more efficient humidity sensors. Meanwhile, the lower vapor permeability for artificial tears in polarized membranes is relevant for biomedical applications, such as ocular sensors for dry eye diagnosis, due to their selective permeability properties.

## Figures and Tables

**Table 1:** Time delay and diffusion coefficient of water and tears samples.

Sample	Water		Tears	
	*t_d_* (s)	*D* (×10^−8^ cm^2^/s)	*t_d_* (s)	*D* (×10^−8^ cm^2^/s)
Polarized	3895.472 ± 68.493	1.782 ± 0.033	26,429.591 ± 587.399	0.263 ± 0.017
Non-polarized	4354.698 ± 90.046	1.629 ± 0.022	2119.471 ± 43.974	3.351 ± 0.012

## Data Availability

The original contributions presented in this study are included in the article. Further inquiries can be directed to the corresponding author.
